# Dual-Energy Computed Tomography, a New Metal Artifact Reduction Technique for Total Hip Arthroplasty: Is There a Light in the Darkness?

**DOI:** 10.3390/jcm14072258

**Published:** 2025-03-26

**Authors:** Andrea Coppola, Luigi Tessitore, Chiara Macina, Filippo Piacentino, Federico Fontana, Andrea Pautasso, Velio Ascenti, Roberto Minici, Domenico Laganà, Tommasa Catania, Giorgio Ascenti, Massimo Venturini, Fabio D’Angelo

**Affiliations:** 1 Diagnostic and Interventional Radiology Unit, Circolo Hospital, ASST Sette Laghi, 21100 Varese, Italy; 2 Department of Science and High Technology, University of Insubria, 22100 Como, Italy; 3 Department of Medicine and Technology Innovation, Insubria University, 21100 Varese, Italy; 4 Orthopedic Surgery Unit, Circolo Hospital, ASST Sette Laghi, 21100 Varese, Italy; 5 Department of Biotechnology and Life Sciences, Insubria University, 21100 Varese, Italy; 6 Department of Diagnostic and Interventional Radiology, Foundation IRCCS Cà Granda-Ospedale Maggiore Policlinico, Via Francesco Sforza 35, 20122 Milan, Italy; 7 Radiology Unit, “Magna Græcia” University, “Renato Dulbecco” University Hospital, Department of Experimental and Clinical Medicine, 88100 Catanzaro, Italy; 8 Diagnostic and Interventional Radiology Unit, BIOMORF Department, University Hospital “Policlinico G. Martino”, 98124 Messina, Italy

**Keywords:** THA, hip arthroplasty, VMI, spectral CT, dual energy, metal artifact

## Abstract

**Background/Objectives**: To evaluate dual-energy computed tomography (DECT) in comparison with conventional CT for periprosthetic bone and surrounding soft tissues in total hip arthroplasty (THA). **Methods**: Two authors independently screened titles and abstracts for eligibility, discussing any disagreements with a third author for final decisions. The articles were categorized into two main groups: those focusing on periprosthetic bone and those on blood vessels or pelvic organs. **Results**: A total of 37 articles were selected to be included in this systematic review. **Conclusions**: Our systematic review reveals significant variability in the use of DECT for periprosthetic bone and soft tissue imaging, due to differences in equipment, protocols, and clinical settings. While many studies indicate that virtual monochromatic imaging (VMI), especially when combined with metal artifact reduction (MAR), improves image quality, there is no consensus on optimal energy levels. Future research should focus on large-scale, multicenter studies with standardized protocols to compare reconstruction techniques, energy levels, and combined MAR-VMI use.

## 1. Introduction

Dual-energy computed tomography (DECT) represents one of the most important technological advancements in medical imaging. By acquiring data at two distinct energy levels (typically 80–140 kVp or equivalents), this technology provides additional information compared to conventional CT, enhancing tissue characterization, disease diagnosis, and optimization of therapeutic pathways. Through specific analysis of the chemical composition and physical properties of materials, DECT finds applications across a wide range of clinical fields. For instance, in oncology, it enables better discrimination between tumor lesions, healthy tissue, and necrotic areas. At the same time, in musculoskeletal radiology, it facilitates the identification and characterization of crystal deposits (such as uric acid in gout). In the cardiovascular field, DECT improves the assessment of atherosclerotic plaques and thrombotic material, while in thoracic radiology, it enhances the detection of pulmonary embolisms and pulmonary perfusion. Equally important is its use in abdominal imaging, where it excels in optimizing the evaluation of liver, pancreatic, and renal lesions, as well as in monitoring responses to oncological treatments [1,2,3,4,5].

In orthopedic settings, DECT offers significant advantages in evaluating both bone and soft tissues given its ability to differentiate materials with similar physical and chemical properties, improving diagnostic accuracy in these complex clinical scenarios. DECT may allow for better differentiation between normal bone, sclerotic bone, and areas of osteolysis. Its ability to separate materials with similar densities but different chemical compositions may improve sensitivity in detecting these changes at an early stage [6].

Differentiating between infection and other conditions (such as mechanical implant failure) is often complex. DECT can highlight fluid collections in periprosthetic soft tissues and help to characterize their composition (e.g., differentiating serous fluid from purulent material). Additionally, iodine mapping can enhance the detection of hyperperfused areas associated with inflammatory or infectious processes [7].

Evaluating periprosthetic soft tissues, such as muscles and ligaments, is often challenging due to artifacts and low resolution in conventional techniques. DECT, with its ability to generate material-specific maps (e.g., separating calcium, urates, and soft tissues), provides better characterization of surrounding soft tissues. This is particularly useful in detecting calcifications, hematomas, or crystal deposits, such as those associated with gout [8].

DECT is valuable not only for diagnosis but also for postoperative monitoring and planning revision surgeries. Its ability to accurately assess residual bone and implant stability contributes to improved clinical outcomes [9].

In summary, DECT represents a significant advancement in the management of periprosthetic pathologies, combining enhanced image quality with a unique capability to characterize materials and tissues, making it an essential tool for radiologists and orthopedic surgeons.

However, orthopedic prostheses (e.g., hip or knee implants) produce metal artifacts that degrade image quality, making it difficult to assess surrounding structures. Only considering total hip replacements (THA), this issue may affect more than 1 million patients per year worldwide [10]. By integrating metal artifact reduction (MAR) algorithms and virtual monoenergetic imaging (VMI), DECT promises to enhance image quality, allowing better visualization of periprosthetic bone and surrounding soft tissues. This may be particularly useful for detecting fractures, osteolysis, or periprosthetic infections [6,11].

The purpose of our systematic review was to critically assess the clinical value of dual-energy CT (DECT) as a novel spectral imaging technology for the evaluation of bone, with a particular focus on its applications in total hip arthroplasty (THA). Specifically, we aimed to determine whether DECT provides meaningful advantages in addressing key challenges in this context, such as the reduction in metal artifacts and the improved visualization of periprosthetic soft tissues.

Additionally, we sought to analyze the perspectives of different authors on the use of virtual monochromatic images (VMIs) generated by DECT, evaluating how many favored VMI as a standalone solution and how many preferred its combination with metal artifact reduction (MAR) techniques. By synthesizing the available evidence, our goal was to evaluate DECT’s potential to enhance diagnostic accuracy and clinical decision-making compared to conventional imaging modalities while identifying areas where its clinical role remains debated or underutilized.

## 2. Materials and Methods

For the literature search in the PubMed database, the search term was as follows: “[1] ((THA) OR (hip arthroplasty)) AND ((VMI) OR (spectral CT) OR (dual energy)); [2] ((VMI) OR (spectral CT) OR (dual energy)) AND (metal artifact) AND (hip)” (THA: Total Hip Arthrosplasty; VMI: “Virtual Monoenergetic Images”).

Two review authors (A.C., L.T.) independently screened the titles and abstracts of articles retrieved by each search to assess eligibility. When a title or abstract could not be excluded with certainty, the full text was evaluated.

The full text of all the eligible articles was then reviewed by the same two authors. We resolved disagreements at any stage of the eligibility assessment process through discussion or by consulting a third author (F.D.A.), and together, we made a final decision.

The article research was supplemented with additional information from the bibliography of the findings of the original search. Only articles that were published in English were selected.

We decided to divide the review of articles into two main categories: articles that address the visualization of periprosthetic bone and articles that address the visualization of blood vessels or pelvic organs. We further divided each category into ex vivo and in vivo articles, distinguishing within each of these subcategories based on the imaging techniques described by the authors as the best for reducing metal artifacts (MAR; VMI; VMI in combination with MAR) or based on the best visualization of specific prosthetic materials and/or organs and/or periprosthetic soft tissues.

## 3. Results

**Figure 1:**
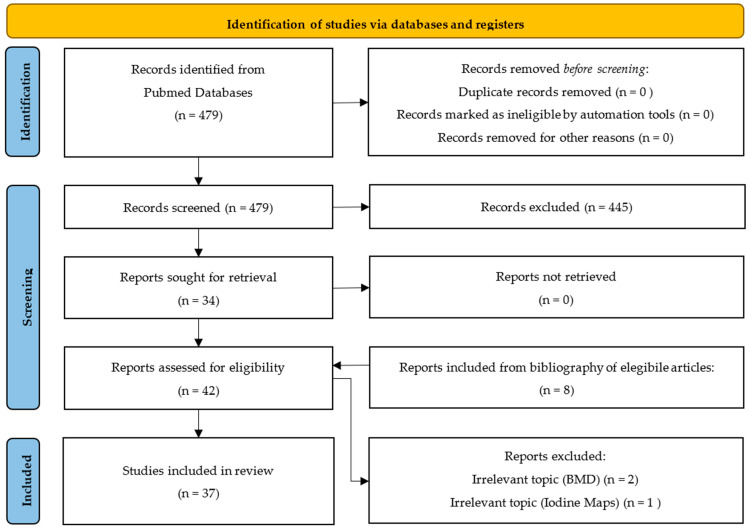
Flowchart of the article selection process.

After thoroughly examining the results, 37 articles were selected to be included in this systematic review (Supplementary Table S1). The flowchart of the article selection process as recommended by the Preferred Reporting Items for Systematic Reviews and Meta-Analyses (PRISMA) [12] is represented in Figure 1. The PRISMA checklist is available in Supplementary Table S2. To strengthen the study, a quality assessment of included studies (QUADAS-2) is provided in Supplementary Table S3.

## 4. Discussion

After thoroughly examining the results, the main techniques reported were conventional imaging (CI), metal artifact reduction (MAR), virtual monochromatic imaging (VMI), and pseudo-monochromatic imaging (PMI). An explanatory table on the main differences among these techniques is provided (Table 1).

### 4.1. Visualization of Periprosthetic Bone 

#### 4.1.1. Ex Vivo

Among the ex vivo studies examining the visualization of the periprosthetic bone, we identified a total of 15 studies, of which 2 dealt with pseudo-monochromatic imaging (PMI) [13,14] (which has never been used in daily clinical practice, but we included these studies in the discussion for completeness), 5 studies favored conventional images (CIs) combined with the metal artifact reduction (MAR) algorithm [15,16,17,18,19], 5 studies favored virtual monochromatic imaging (VMI) combined with the MAR algorithm [11,20,21,22,23], and 3 studies favored VMI alone [24,25,26]. 

Of the two studies dealing with PMI, one still favored VMI combined with the MAR protocol over the PMIs in the study [13], so we can state that, in total, six studies preferred these images over other modalities. The other study, which favored PMI combined with the MAR protocol for visualizing the periprosthetic area, also only compared such images with conventional ones, whether or not combined with the MAR protocol, without studying the role of VMI compared to this method [14]. 

In the category of five ex vivo studies favoring CI in combination with MAR, three were cadaveric studies, with a very small number of cadavers or cadaveric samples (six cadavers [16], two samples [19], and one fresh cadaver pelvis [15]); two were phantom studies, one on a phantom pelvis with unilateral or bilateral THA [18] and the other on a prosthetic phantom of unilateral THA (styrofoam, polyethylene, and water) [17]. Two out of these five studies considered not only THA but also other types of prostheses and body metallic implants (e.g., dental or spinal) [16,19]. Furthermore, three out of these five studies performed both a quantitative analysis using specific software and a qualitative visual analysis with evaluation using image satisfaction scales (five-point Likert scale) conducted by radiologists [16,18,19]. Finally, only two studies in this group identified an optimal keV threshold that is recommended by the authors for reducing periprosthetic metal artifacts: 140 keV in one case [17] and variable according to the material of the implant (bilateral titanium THA: 143 keV; unilateral Fe THA: 94 keV; bilateral Fe THA: 83 keV; unilateral Ti THA: 107 keV) [18]. Out of these studies, a special consideration must be given to the work by Huflage H. and Colleagues [19], in which conventional imaging was acquired in combination with tin (_50_Sn)-filtered imaging with 100 and 150 kVp (SOMATOM Force, Siemens Healthineers, Munich, Germany), and this technique gave the best image quality in their opinion.

Of these five ex vivo studies in favor of VMI in combination with MAR, only one was a cadaveric study conducted on extra-articulated prostheses (nine THAs, of which six were uncemented and three cemented) [20]. The others were phantom studies with bilateral and/or unilateral prostheses [11,21,22,23]. 

Thus, despite the limited number of samples, these were still relatively homogeneous as they examined only total hip prostheses and no other types of prostheses or metallic implants. All these studies reported quantitative analysis using software; one of them [20] also included a qualitative visual analysis with an evaluation using a five-point Likert scale performed by two radiologists and three orthopedic surgeons. All these five studies recommend an optimal keV threshold for reducing periprosthetic metallic artifacts, but there is no agreement between the proposed values: ranging from 160 to 190 keV in one case [20], 110 keV without the MAR algorithm for bilateral titanium prostheses and 110 keV with the MAR algorithm for cemented cobalt prostheses, from 90 to 130 keV for cobalt-only prostheses and from 90 to 110 keV for stainless steel prostheses in another case [11], 130 keV in a third case [21], less than 100 keV for titanium prostheses and greater than or equal to 100 keV for those in steel [23], and finally 110 keV in the last case [22]. 

Only three studies examined the different metallic compositions of the prostheses and indicated an optimal keV threshold for evaluating periprosthetic bone based on the prosthetic metallic composition. Two studies were in favor of VMI in combination with MAR [11,23], while one was only in favor of CI in combination with MAR [18], although the latter study did not exanimate VMI associated with the MAR protocol but only standalone VMI. 

Finally, the three ex vivo studies in favor of standalone VMI were all performed on prosthetic phantoms, examining total bilateral or unilateral hip prostheses [24,25] or hip prosthesis stems [26]. All of these three studies performed only quantitative analysis using dedicated software, omitting qualitative visual analysis, and obtained recommended KeV threshold values for better periprosthetic visualization, without concordance among recommended values, which were 130 keV in one case [24], 150 keV in the second study [25], and ranging from 70 to 140 keV in the last case [26]. 

#### 4.1.2. In Vivo 

Among the in vivo studies examining the visualization of periprosthetic bone, we found 13 studies, of which 11 were retrospective and 2 prospective, and out of these, only 1 supported CI associated with the MAR algorithm [27], 5 supported VMI in combination with MAR [28,29,30,31,32], and 7 studies favored VMI alone [33,34,35,36,37,38,39]. 

As previously mentioned, the only in vivo study in this group favoring CI in combination with MAR is the one from Yoo HJ and colleagues [27], a retrospective study on 47 patients with 58 THAs (11 bilateral and 36 unilateral). In this study, both a quantitative analysis using dedicated software and a qualitative visual analysis using a five-point Likert scale were performed by two radiologists with 5 and 10 years of experience, respectively. The authors preferred CI + MAR over VMI, particularly for the evaluation of periprosthetic soft tissues rather than periprosthetic bone, for which high-KeV VMIs are described as very advantageous. The suggested optimal threshold for periprosthetic soft tissue visualization ranges from 120 to 200 KeV, while a 200 KeV threshold is suggested for periprosthetic bone. A major limitation of this study, beyond its retrospective nature and the limited number of patients, is that it does not consider VMIs associated with the MAR protocol, focusing only on VMIs alone. 

The five in vivo studies favoring VMI in combination with MAR [28,29,30,31,32] were all retrospective, recruiting a number of patients ranging from 24 to 46 with both unilateral and bilateral THAs. In two studies, other metallic prosthetic implants were included (dental implants in one case [31], and knee, shoulder, radial head, and ankle arthroplasty, tumor prosthesis, osteosyntheses with plates or screws in the other one [32]). All studies conducted both quantitative analyses using specific analysis software and qualitative visual analyses using a five-point Likert scale by specialized radiologists. Three of these studies identified an optimal KeV range for visualizing periprosthetic bone and minimizing metal artifacts, with substantial variability among identified values: 120–140 KeV in one case [28], 140–200 KeV in the second study [29], and 160–200 keV in the last one [30]. 

Of the seven in vivo studies favoring VMI alone for visualizing periprosthetic bone, five were retrospective [33,34,35,38,39] and two prospective [36,37]. The median number of patients recruited was 27, ranging from 12 to 178. In one case [35], a phantom was also used. Three of these studies examined only THAs, while the remaining four studies included various metallic prosthetic implants, including THAs. All of the seven studies performed both quantitative analyses using specific software and qualitative visual analyses using image rating scales (five-point Likert scale) by specialized radiologists. Of these studies, only one study [37] did not describe an optimal keV threshold for visualizing periprosthetic bone; the remaining six studies did so with a certain agreement (proposed values: 110 keV [33]; 113 keV or better; a range of 100–130 keV [35]; 130 keV [38]; 140 keV [34]; 155 keV [39]). 

The main limitations of these studies, beyond the small number of patients (except for Foti G. and colleagues’ study [39], which retrospectively enrolled 178 patients), include the variability in prosthetic implants tested. 

Additionally, some authors favoring VMI alone did not test CI with or without the MAR protocol [34,38]. Even the two prospective studies, despite their design, suffer from a small number of patients and prostheses analyzed, as well as the inclusion of various metallic implants and not only THAs [36,37]. 

Out of these studies, we highlight the one from Magarelli N and colleagues [37], who specifically used a Siemens proprietary spectral reconstruction (“Opt Kev, Siemens HealthCare, Forchheim, Germany) in which the software automatically extrapolates the optimal KeV value from VMI reconstructions.

In conclusion, for periprosthetic bone visualization, we found a total of 26 studies. Out of them, six favored CI in combination with MAR (5 ex vivo, 1 in vivo), ten favored VMI in combination with MAR (5 ex vivo, 5 in vivo), and the last ten favored VMI alone (3 ex vivo, 7 in vivo). For the optimal keV threshold for visualizing periprosthetic bone, wide discrepancies between different authors were identified. The most congruent group of authors, favoring VMI alone, suggest a keV threshold between 100 and 155 KeV. 

### 4.2. Visualization of Pelvic Organs/Periprosthetic Soft Tissue and Vascularization 

#### 4.2.1. Ex Vivo 

Among the ex vivo studies examining the visualization of pelvic organs, soft tissues, and periprosthetic vascularization, we identified only two studies, both prospective: one favoring VMIs associated with the MAR protocol [40] and one favoring VMIs alone [41]. 

The first one, by Kovacs DG and colleagues [40], was conducted on three phantoms containing various prosthetic implants (dental, spinal, and hip implants), while the second by Filograna L and colleagues [41] was a cadaveric study on twenty specimens, also with various metallic prosthetic implants. Both studies performed both a quantitative analysis using specific software and a qualitative visual analysis using image rating scales (five-point Likert scale) conducted by specialized radiologists (in one study oncologists also evaluated the images [40]). Both studies identified a quite similar optimal keV threshold for visualizing periprosthetic soft tissues, 130 keV in one case [40], and 137.6 ± 4.9 keV in the second one [41]. 

The main limitations of these studies, beyond their ex vivo nature, include the analysis of different metallic implants and the small number of specimens analyzed. Additionally, the study by Filograna L [41] does not consider the MAR protocol at all. 

#### 4.2.2. In Vivo 

Among the in vivo studies examining the visualization of pelvic organs, soft tissues, and periprosthetic vascularization, we identified a total of seven studies, six retrospective and one prospective. Among these, one favored CI in combination with MAR [42], four favored VMI in combination with MAR [43,44,45,46], and two favored VMI alone [47,48]. 

The only in vivo study in this group favoring CI in combination with MAR is by Wichtmann HM and colleagues [42], a retrospective study involving 102 patients with 71 THA (26 bilateral and 45 unilateral) and 31 metallic spinal implants. The study included both quantitative analyses using dedicated software and qualitative visual analysis with evaluation through image rating scales (e.g., five-point Likert scale) by specialized radiologists. The authors favored CI in combination with MAR (described as “MixedIMAR”) because, in their opinion, it provides better image quality. However, they also note that low-keV images (50 KeV) can be used in conjunction with the iMAR protocol to enhance vascular contrast while reducing metallic artifacts compared to non-iMAR images. Conversely, high-keV VMI can quantitatively reduce metallic artifacts but does not improve overall image quality, according to the author. The study identifies an optimal keV threshold of approximately 120 keV for visualizing periprosthetic soft tissues. Unlike the study by Yoo HJ [27], which did not consider VMI associated with the MAR protocol, this study overcomes that limitation and stands out in our review as the only in vivo study favoring conventional images with MAR over VMI in combination with MAR. Despite the substantial number of patients analyzed, this study is not without limitations, such as its retrospective nature and the heterogeneity of the tested materials (including both THA and spinal implants). 

The four in vivo studies favoring VMI in combination with MAR, three of which were retrospective [43,44,45] and one prospective [46], included varying numbers of patients, ranging from 30 to 80. In two cases, only THA patients were considered, and in the other two, spinal prostheses [45] and various metallic materials [46] were also considered. All four studies performed both quantitative analyses using dedicated software and qualitative visual analysis with evaluation through image rating scales (e.g., five-point Likert scale) by specialized radiologists. Only three of these studies identified an optimal threshold for visualizing pelvic organs and periprosthetic soft tissues: 80 keV in one case [43], 77 keV in the second case [44], and 140 keV in the last one [45].

The two in vivo studies favoring VMI alone were both retrospective, analyzing 35 [47] and 39 [48] patients, respectively, with various metallic body implants in both cases. Both studies conducted quantitative analysis using dedicated software and qualitative visual analysis with evaluation through image rating scales (e.g., five-point Likert scale) by specialized radiologists. Both studies reported an optimal keV threshold for visualizing pelvic and periprosthetic soft tissues with similar findings (140 keV [47], and 130 keV [48]). Beyond the small number of patients and the high variability of prosthetic implants analyzed, a significant limitation of these studies is their failure to test all acquisition methods: one study [47] focuses solely on VMI and does not consider conventional images or the MAR protocol, while the second one [48] omits only the MAR protocol. 

In conclusion, regarding the visualization of pelvic organs, soft tissues, and periprosthetic vascularization, we found a total of nine studies, with one in vivo favoring CI in combination with MAR, one ex vivo and four in vivo studies favoring VMI in combination with MAR, and one ex vivo and two in vivo studies favoring VMI alone.

## 5. Conclusions

Our extensive systematic review highlights a significant degree of heterogeneity in the current literature regarding the application of dual-energy CT (DECT) in periprosthetic bone and soft tissue imaging. This variability arises from differences in imaging equipment, protocols, reconstruction techniques, and clinical settings employed across studies. Some authors focused exclusively on metal artifact reduction (MAR), others on virtual monochromatic imaging (VMI), and a subset considered a combination of these techniques, further contributing to the inconsistency in findings.

Despite these challenges, certain trends can be observed. The majority of studies suggest that VMI, either used alone or in combination with MAR, offers notable advantages in improving image quality. Specifically, high-energy VMI (at higher keV levels) has been associated with better visualization of periprosthetic bone, while low-energy VMI appears to enhance the assessment of soft tissues. However, several limitations remain evident. Firstly, there is a lack of unified thresholds; at present, no universally agreed-upon keV thresholds have been established for optimal imaging, as the “ideal” energy level varies depending on the specific clinical question and the imaging system used. In our opinion, the integration of VMI with MAR remains indispensable in most clinical scenarios to minimize metal artifacts and improve diagnostic accuracy, particularly in highly artifact-prone settings such as total hip arthroplasty (THA). A comparative table of advantages and disadvantages of MAR, VMI, and PMI is available in Table 2.

Given these observations, we conclude that the combination of VMI and MAR currently represents the most effective strategy for reducing metal artifacts in patients with hip arthroplasty. Specifically, for periprosthetic bone evaluation, the combined use of VMI and MAR is recommended, with an optimal energy range between 110 and 155 keV, depending on the prosthetic material. For soft tissue and pelvic organ evaluation, optimal VMI energy values generally range between 77 and 140 keV; MAR integration is recommended in cases of high metal interference.

Future research should prioritize the design and execution of large-scale, multicenter studies utilizing diverse DECT systems. These studies should focus on the following: (1) standardization of protocols establishing uniform parameters for the use of VMI, MAR, and PMI in different types of prostheses and materials; (2) direct comparisons between DECT machines, evaluating differences between various manufacturers to optimize clinical use; (3) integration of artificial intelligence, developing machine learning algorithms to improve automatic keV selection and reduce artifacts; (4) large-scale multicenter studies, comparing different approaches in large patient populations to establish more robust guidelines; (5) expanding PMI clinical use, assessing its impact on artifact reduction across various implant types and anatomical regions. Such efforts are critical in establishing evidence-based guidelines for the effective clinical use of DECT in this challenging field.

In summary, while DECT shows promise for advancing the evaluation of periprosthetic bone and soft tissues, more robust and standardized research is needed to fully define its potential and optimize its clinical applications.

## Figures and Tables

**Table 1:** Explanatory table on CI, MAR, VMI, and PMI.

Technique	Description	Main Clinical Applications
**Conventional Imaging (CI)**	Standard imaging obtained with conventional CT without energy modifications.	Standard CT scan. General evaluation of anatomical structures; however, it may be limited by the presence of metal artifacts and suboptimal contrast in certain applications.
**Metal Artifact Reduction (MAR)**	Algorithms that are used to improve CT image quality in patients with metalware. MAR algorithms can be associated with either CI, VMI, or other acquisition techniques.	Enhancement of the visualization of anatomical structures adjacent to metalware. MAR algorithms are available on most CT scanners.
**Virtual Monochromatic Imaging (VMI)**	Images generated at different energies (keV) using DECT to reduce artifacts and improve contrast.	Bone evaluation, metal artifact reduction, periprosthetic structure assessment, enhanced contrast conditions in oncology and vascular imaging. VMI requires DECT scanner.

**Table 2:** Comparative table of advantages and disadvantages of MAR, VMI, and PMI.

Technique	Advantages	Disadvantages
**Metal Artifact Reduction (MAR)**	Significantly reduces metal artifacts, improving the visualization of bones and soft tissues; it is compatible with a wide range of CT scanners.	It may introduce new distortions or secondary artifacts; effectiveness may vary depending on the type of metal implant and the specific technique used.
**Virtual Monochromatic Imaging (VMI)**	Allows selection of the optimal energy level (keV) to improve image quality and reduce artifacts; may reduce the amount of contrast medium required or radiation dose.	The optimal keV selection varies depending on the type of prosthesis and diagnostic objective; it requires expertise in interpretation; effectiveness may be influenced by the presence of significant metal artifacts.
**Pseudo-Monochromatic Imaging (PMI)**	Reduces beam hardening and metal artifacts in certain cases.	Reduced contrast-to-noise ratio (CNR); limited effectiveness with intense metal artifacts.

## Data Availability

Data derived from public domain resources (Pubmed).

## Supplementary Materials

The following supporting information can be downloaded at: https://www.mdpi.com/article/10.3390/jcm14072258/s1, Table S1: List of the articles. Table S2: PRISMA checklist. Table S3: QUADAS-2.

## Abbreviations

The following abbreviations are used in this manuscript:

CI	Conventional images
CT	Computed tomography
DECT	Dual-energy CT
Fe	Iron
iMAR	Iterative metal artifact reduction
KeV	Kilo-electronVolts
MAR	Metal artifact reduction
PMI	Pseudo-monochromatic imaging
Sn	Tin (Stannum)
THA	Total hip arthroplasty
Ti	Titanium
VMI	Virtual monochromatic imaging
