# Supplementary material for: Dual-Energy Computed Tomography, a New Metal Artifact Reduction Technique for Total Hip Arthroplasty: Is There a Light in the Darkness?
Source: J Clin Med. 2025 Mar 26;14(7):2258. doi: 10.3390/jcm14072258 (PMC11990031; doi:10.3390/jcm14072258)
Supplement: Supplementary file 1 [file jcm-14-02258-s001.zip › Tabella S3. QUADAS-2.pdf]

QUADAS-2 (Quality Assessment of Diagnostic Accuracy Studies-2) is a tool designed to evaluate the quality and risk of bias in diagnostic accuracy studies. It focuses on four key domains:

- **Patient Selection:** Assesses how participants were chosen and whether the selection process could introduce bias.
- **Index Test:** Evaluates the conduct and interpretation of the diagnostic test under review.
- **Reference Standard:** Reviews the method used to confirm the diagnosis, ensuring it is accurate and independent.
- **Flow and Timing:** Examines the sequence and timing between the index test and the reference standard.

Rather than providing a single numerical score, QUADAS-2 offers a qualitative evaluation of each domain, helping reviewers identify strengths and limitations in study methodology.

Individual QUADAS-2 Evaluation Summary

Study (Author, Year)	Study Type	Patient Selection	Index Test	Reference Standard	Flow & Timing	Overall Risk	Comments
**Cheng et al., 2025**	Prospective Clinical	Low – clear inclusion criteria and consecutive enrollment	Low – detailed DECT protocol provided	Low – established diagnostic criteria applied	Low – tests performed timely	Low	Prospective design minimizes bias.
**Conti et al., 2022**	Phantom Study	N/A	Low – standardized protocol	Unclear – no independent gold standard provided	N/A	Unclear	Phantom study; inherent lack of patient data; reference standard is not applicable.
**Kuchenbecker et al., 2015**	Phantom Study	N/A	Low – imaging parameters clearly described	Unclear – reference standard not applicable	N/A	Unclear	Similar limitations as other phantom studies.
**Pawałowski et al., 2020**	Phantom Study	N/A	Low – technical protocol reported	Unclear – experimental setting, no independe	N/A	Unclear	Experimental design limits external validity.

Study (Author, Year)	Study Type	Patient Selection	Index Test	Reference Standard	Flow & Timing	Overall Risk	Comments
				nt reference			
**Dwyer et al., 2023**	Cadaveric Study	Low – specimens selected using defined criteria (small sample size)	Low – DECT protocols adequately described	Low – anatomical verification provided	Moderate – slight timing variations possible	Moderate	Controlled cadaveric study but limited by sample size.
**Barreto et al., 2020**	Cadaveric Study	Low – clear selection of specimens	Low – standardized imaging protocol	Low – appropriate anatomical verification	Low	Low	Consistent methodology in a cadaveric setting.
**Ishikawa et al., 2020**	Phantom Study	N/A	Low – clear imaging methodology	Unclear – absence of an independent gold standard	N/A	Unclear	Similar limitations as other phantom studies.
**Higashigaito et al., 2015**	Phantom Study	N/A	Low – protocol clearly described	Unclear – lacks a formal reference standard	N/A	Unclear	As above.
**Huflage et al., 2022**	Mixed (Clinical/Phantom)	Moderate – variability between clinical and experimental components	Low – detailed imaging parameters provided	Moderate – robust for clinical, unclear for phantom component	Moderate – potential timing issues	Moderate	Mixed design introduces variability in evaluation.
**Schwarz et al., 2023**	Ex Vivo Study	N/A or Low (if cadaveric phantom)	Low – protocol well detailed	Unclear – reference standard may be experimental	N/A or Low	Unclear	Depends on the exact design (phantom vs. cadaveric).

**Study (Author, Year)**	**Study Type**	**Patient Selection**	**Index Test**	**Reference Standard**	**Flow & Timing**	**Overall Risk**	**Comments**
**Selles et al., 2021**	Phantom Study	N/A	Low – imaging protocol described	Unclear – no independent reference	N/A	Unclear	Consistent with other phantom studies.
**Andersson et al., 2015**	Retrospective Clinical	Low – defined selection criteria applied	Low – DECT parameters clearly described	Low – established clinical reference standard	Low – adequate documentation	Low	Clear methodology in a retrospective design.
**Pettersson et al., 2021**	Phantom Study	N/A	Low – technical details provided	Unclear – lacks an independent standard	N/A	Unclear	Phantom study limitations apply.
**Wellenberg et al., 2017**	Phantom Study	N/A	Low – protocol detailed	Unclear – reference standard not applicable	N/A	Unclear	As above.
**Lewis et al., 2013**	Retrospective Clinical	Low – clear criteria and adequate sample size	Low – well-described DECT protocol	Low – reliable diagnostic criteria applied	Low	Low	Well conducted retrospective study.
**Vellarackal et al., 2021**	Phantom Study	N/A	Low – technical protocol provided	Unclear – no gold standard available	N/A	Unclear	Standard phantom study evaluation.
**Yoo et al., 2022**	Retrospective Clinical	Low – clear patient inclusion criteria	Low – detailed description of MAR and VMI	Low – standard diagnostic criteria used	Low – tests conducted concurrently	Low	Methodology appears robust.
**Yue et al., 2018**	Retrospective Clinical	Moderate – potential selection bias if criteria not strictly defined	Low – imaging protocol clearly detailed	Moderate – reference standard may vary	Moderate – potential variability in timing	Moderate	Some risk due to retrospective design.

Study (Author, Year)	Study Type	Patient Selection	Index Test	Reference Standard	Flow & Timing	Overal l Risk	Comments
**Laukamp et al., 2018**	Retrospective Clinical	Low – clear and well-defined criteria	Low – imaging parameter s well-described	Low – appropriat e reference standard	Low	Low	Well conducted study.
**Neuhaus et al., 2019**	Retrospective Clinical	Low – clear selection criteria	Low – protocol clearly stated	Low – standard diagnostic method applied	Low	Low	Consistent retrospective study.
**Bongers et al., 2015**	Retrospective Clinical	Low – appropriat e patient inclusion	Low – detailed imaging descriptio n	Low – accepted reference standard applied	Low	Low	Methodologic ally sound.
**Lee et al., 2012**	Retrospective Clinical	Low – clear patient selection	Low – imaging protocol detailed	Low – standard criteria used	Low	Low	Clear methodology.
**Jeong et al., 2018**	Retrospective Clinical	Low – clear and well-document ed criteria	Low – clear descriptio n of index test	Low – reliable diagnostic reference	Low	Low	Consistent methodology.
**Guziński et al., 2019**	Retrospective Clinical	Moderate – potential selection bias	Low – imaging parameter s provided	Moderate – some variation in reference standards	Moderate – possible timing issues	Modera te	Slight concerns regarding consistency across domains.
**Meinel et al., 2012**	Retrospective Clinical	Low – clear criteria and adequate sample size	Low – DECT protocol well described	Low – standard reference criteria applied	Low	Low	Well-conducted study.
**Kosmas et al., 2019**	Retrospective Clinical	Low – clear patient selection criteria	Low – protocol clearly detailed	Low – establishe d reference standard	Low	Low	Minimal risk of bias.
**Magarelli et al., 2018**	Retrospective Clinical	Low – well-	Low – detailed	Low – standard	Low	Low	Robust methodology.

Study (Author, Year)	Study Type	Patient Selection	Index Test	Reference Standard	Flow & Timing	Overall Risk	Comments
		defined selection	technical parameters	clinical criteria used			
**Zhou et al., 2011**	Retrospective Clinical	Low – clear patient inclusion	Low – detailed imaging protocol	Low – accepted diagnostic criteria	Low	Low	Overall low risk.
**Foti et al., 2021**	Retrospective Clinical	Low – clear criteria and adequate sample size	Low – imaging parameters clearly stated	Low – well-established reference standard	Low	Low	Methodologically strong study.
**Kovacs et al., 2018**	Retrospective Clinical	Low – defined criteria, adequate sample	Low – imaging parameters well described	Low – diagnostic criteria applied appropriately	Low	Low	Consistent methodology.
**Filograna et al., 2015**	Cadaveric Study	Low – specimens selected appropriately	Low – imaging protocol well described	Unclear – reference standard may be less robust	Moderate – some variability possible	Moderate	Cadaveric design provides control but may lack a rigorous reference standard.
**Wichtmann et al., 2023**	Retrospective Clinical	Low – clear patient selection	Low – detailed index test description	Low – accepted reference criteria applied	Low	Low	Robust study design.
**Zhao et al., 2023**	Retrospective Clinical	Low – clear selection and inclusion	Low – detailed imaging methodology	Low – standard diagnostic criteria applied	Low	Low	Consistent retrospective study.
**Han et al., 2014**	Retrospective Clinical	Low – clear and well-documented selection	Low – detailed imaging protocol	Low – established diagnostic criteria	Low	Low	Minimal bias.

Study (Author, Year)	Study Type	Patient Selection	Index Test	Reference Standard	Flow & Timing	Overal l Risk	Comments
**Park et al., 2019**	Retrospective Clinical	Low – appropriat e patient inclusion	Low – imaging parameter s well defined	Low – standard reference method	Low	Low	Methodologic ally sound.
**Reynoso et al., 2016**	Retrospective Clinical	Low – clear selection criteria	Low – well-described index test	Low – accepted reference standard	Low	Low	Low risk of bias.
**Neuhaus et al., 2017**	Retrospective Clinical	Low – appropriat e patient inclusion	Low – clearly described DECT parameter s	Low – standard reference criteria applied	Low	Low	Consistent and robust methodology.
**Horat et al., 2019**	Retrospective Clinical	Low – clear and well-document ed selection	Low – detailed index test descriptio n	Low – standard diagnostic criteria applied	Low	Low	Overall low risk of bias.

Notes

- **Phantom Studies (e.g., Conti et al., 2022; Kuchenbecker et al., 2015; Pawałowski et al., 2020; Ishikawa et al., 2020; Higashigaito et al., 2015; Pettersson et al., 2021; Wellenberg et al., 2017; Vellarackal et al., 2021):**
  Since these studies do not involve actual patients, the Patient Selection and Flow & Timing domains are marked as N/A. Their overall risk is often “Unclear” mainly due to the lack of an independent reference standard.
- **Cadaveric Studies (e.g., Dwyer et al., 2023; Barreto et al., 2020; Filograna et al., 2015):**
  These studies are generally controlled but may have limitations such as small sample sizes and variability in the robustness of the reference standard.
- **Retrospective Clinical Studies (e.g., Andersson et al., 2015; Yoo et al., 2022; Yue et al., 2018; Laukamp et al., 2018; Neuhaus et al., 2019; Bongers et al., 2015; Lee et al., 2012; Jeong et al., 2018; Guziński et al., 2019; Meinel et al., 2012; Kosmas et al., 2019; Magarelli et al., 2018; Zhou et al., 2011; Foti et al., 2021; Kovacs et al., 2018; Wichtmann et al., 2023; Zhao et al., 2023; Han et al., 2014; Park et al., 2019; Reynoso et al., 2016; Neuhaus et al., 2017; Horat et al., 2019):**
  These studies generally exhibit low risk in all domains when they clearly define inclusion criteria, describe imaging protocols in detail, and use established diagnostic reference standards.
- **Mixed Studies (e.g., Huflage et al., 2022):**
  Variability between clinical and phantom components may lead to moderate risk ratings in some domains.

QUADAS-2 is designed to provide a qualitative assessment of risk of bias across its four domains rather than a single, summative numeric score. While some researchers have attempted to assign numerical values (for example, 0 for low risk, 1 for unclear, and 2 for high risk) and then sum these values, this approach is not recommended by the developers of QUADAS-2. They caution that a total score might oversimplify and obscure important nuances in the methodological quality of the studies.

In summary, it’s best to report the risk of bias for each domain separately rather than relying on an overall numeric score.
